# In Vivo Expression of MHC Class I Genes Depends on the Presence of a Downstream Barrier Element

**DOI:** 10.1371/journal.pone.0006748

**Published:** 2009-08-26

**Authors:** Helit Cohen, Palak Parekh, Zeynep Sercan, Aparna Kotekar, Jocelyn D. Weissman, Dinah S. Singer

**Affiliations:** Experimental Immunology Branch, Center for Cancer Research (CCR), National Institutes of Health (NIH), Bethesda, Maryland, United States of America; Centre National de la Recherche Scientifique, France

## Abstract

Regulation of MHC class I gene expression is critical to achieve proper immune surveillance. In this work, we identify elements downstream of the MHC class I promoter that are necessary for appropriate *in vivo* regulation: a novel barrier element that protects the MHC class I gene from silencing and elements within the first two introns that contribute to tissue specific transcription. The barrier element is located in intergenic sequences 3′ to the polyA addition site. It is necessary for stable expression *in vivo*, but has no effect in transient transfection assays. Accordingly, in both transgenic mice and stably transfected cell lines, truncation of the barrier resulted in transcriptional gene silencing, increased nucleosomal density and decreased histone H3K9/K14 acetylation and H3K4 di-methylation across the gene. Significantly, distinct sequences within the barrier element govern anti-silencing and chromatin modifications. Thus, this novel barrier element functions to maintain transcriptionally permissive chromatin organization and prevent transcriptional silencing of the MHC class I gene, ensuring it is poised to respond to immune signaling.

## Introduction

Major histocompatibility complex class I (MHC I) molecules provide immune surveillance against intracellular pathogens [1]. MHC class I genes are ubiquitously expressed, but in a tissue-specific manner [2]. Their expression is regulated primarily at the transcriptional level and can be modulated both positively and negatively by different stimuli [3]–[6]. Regulation of MHC class I expression level is crucial for proper immune responses. Thus, over-expression of MHC class I is correlated with induction of several autoimmune diseases [7]–[12]. Conversely, MHC class I down-regulation is associated in many cancers with disease progression, therapy resistance and reduced survival [13]–[15]. Down-regulation of MHC class I surface expression is also observed in cells infected with HIV-1 [16], [17] or transformed by oncogenic viruses [18], [19], often resulting from epigenetic alterations across MHC class I genes, including histones deacetylation and DNA hypermethylation [19]–[21]. Taken together, these observations emphasize the importance of understanding the mechanisms that regulate MHC class I expression and epigenetic integrity.

We previously reported the generation of transgenic mice carrying MHC class I gene, PD1. The PD1 transgene is a 9 Kb genomic DNA fragment that contains 4 Kb of swine MHC class I coding sequences, 1 Kb of sequences 5′ to the transcription start site (TSS) and 4 Kb downstream of the polyadenylation site. In mice, the PD1 transgene displays patterns of expression and cytokine responses that parallel those of the endogenous classical MHC class I gene [22], suggesting that 1) the short 9 Kb sequence contains the full regulatory unit for proper expression of the MHC class I gene and 2) regulatory mechanisms are fully conserved across species [23].

Detailed *in vivo* and *ex vivo* molecular analyses of the promoter of MHC class I gene have identified a series of regulatory DNA sequence elements 5′ to the coding sequences that serve to establish proper patterns of gene expression [24], [25]. Tissue-specific expression is achieved through the combined effects of a promoter-distal complex regulatory element located between -700 and –800 bp, and a series of promoter-distal elements [2], [26], [27]. Hormone/cytokine signaling is mediated through a series of promoter-proximal elements, located between –68 and –500 bp [3], [5]. The cognate DNA-binding transcription factors that interact with these promoter-proximal and -distal DNA sequence elements have been identified [4], [6]. At the core promoter, differences in Pol II occupancy correlate with differences in rates of transcription (J. Weissman and D. Singer, manuscript in preparation, 2009).

Despite the detailed understanding of the role of promoter DNA sequence elements and transcription factors in establishing tissue-specific levels of MHC class I expression and hormone/cytokine-mediated responses, little is known about either the role of downstream sequence elements or of chromatin structure in the regulation of this gene family. Recently, we reported the surprising finding that chromatin structure does not actively regulate transcription of the class I gene: nucleosomal occupancy and positioning are indistinguishable in tissues that differ in class I expression levels by an order of magnitude and do not vary upon either induction or inhibition of transcription. Rather, the chromatin organization functions to keep the core promoter poised and accessible for transcription, allowing rapid activation of the gene without chromatin remodeling [28]. These findings are consistent with the stable expression of the PD1 transgene and suggested the possible existence of a barrier element within the 9 Kb genomic PD1 sequence that functions to maintain an open chromatin structure.

In the present study, we have examined the role of sequences downstream of the promoter in regulating MHC class I gene expression. We demonstrate that the promoter alone is not sufficient for proper regulation of PD1 expression *in vivo*. Rather, stable PD1 expression and transcription-permissive chromatin organization depend on the presence of a 3′ barrier element downstream of the PD1 gene. Distinct barrier element sequences are necessary to prevent silencing and to maintain an open chromatin organization across the MHC class I gene both in transgenic mice and in stably transfected cells. In addition, sequence elements within the introns contribute to the proper regulation of tissue specific expression.

## Results

### Sequences downstream of the promoter of the MHC class I gene, PD1, are required for proper *in vivo* expression

**Figure 1:**
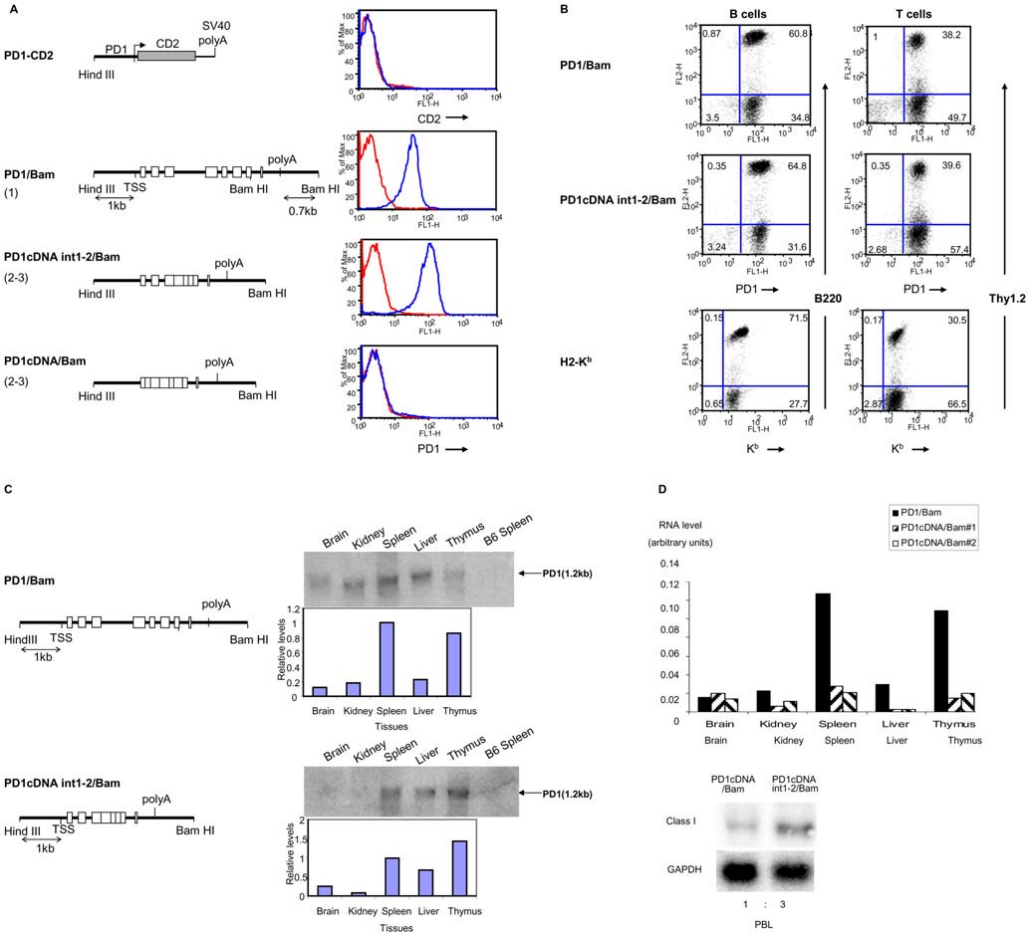
Sequences downstream of the MHC I promoter are required for proper surface expression on PBL *in-vivo*. (A) FACS profiles (right panels) of PD1 or CD2 surface expression on PBL of mice carrying the transgenes with the 1 Kb PD1 promoter ligated to the huCD2 reporter (PD1-CD2); full length PD1 (PD1/Bam) transgene or PD1 cDNA with the 3′ Bam HI fragment (PD1cDNA/Bam), cDNA containing introns 1 and 2 with a 3′ Bam HI fragment (PD1cDNAint1-2/Bam). (diagramed on left panels). PBL were stained with either anti-PD1 or anti-CD2 antibody, as described in Materials and Methods. Transgene copy numbers are indicated in parenthesis. Red curves represent staining of negative control C57/BL6 mice with the relevant antibody. Profiles are representative of all of the founders of each of the transgenic lines. (B) MHC class I expression patterns on B and T cells. Surface expression of MHC class I on B and T cells derived from mice transgenic for PD1/Bam and PD1cDNAint1-2/Bam was assessed by dual staining with anti-PD1 antibody and B220 (B cells) or Thy1.2 (T cells). The pattern of endogenous mouse MHC class I, K^b^, expression on the PBL from PD1/Bam mice was determined by staining with an anti- K^b^ antibody. The K^b^ and PD1 antibodies do not cross-react (data not shown). The results are representative of multiple independent experiments. (C) PD1 RNA expression in PD1cDNAint1-2/Bam mice parallels that of PD1/Bam in different tissues. PD1 RNA levels in tissues of transgenic mice were determined by both Northern analysis using a probe that spans exons 2–3 and that only minimally cross-hybridizes with endogenous class I sequences. Relative levels of expression were quantitated by normalizing with an 18S RNA control probe (graphs below northern). This experiment is representative of three independent experiments. qPCR analyses are shown in Figure S1. (D) PD1 expression in PD1cDNA/Bam transgenic mice is aberrant. RNA from PBL (right panel) or various tissues was probed with a class I probe, by Northern as in (C) and normalized with GAPDH. The relative levels of expression in two individual PD1cDNA/Bam mice are shown in the left panel.

To determine whether the PD1 promoter contains all the regulatory elements necessary for normal *in vivo* expression, transgenic mice were generated from a construct containing the 1 Kb PD1 promoter segment ligated to a human CD2 reporter [29]. In contrast to the genomic PD1 transgenic lines, none of the nine independent PD1-CD2 lines expressed the CD2 reporter transgene (Figure 1A, top panel; Table 1). Similarly, when the class I promoter was ligated to a GFP reporter, none of 6 independent transgenic lines generated from this construct expressed GFP protein or RNA (J.Lovchick and D. Singer, unpublished observations). Thus, although the extended 1 Kb PD1 promoter is functional and regulated in transient transfection assays [3], [24], it is insufficient to direct stable class I transcription in transgenic mice, suggesting that sequences downstream of the transcription start site are necessary *in vivo*.

**Table 1:** MHC class I promoter driven expression of the CD2 transgene depends on the presence of intergenic sequences 3′ to the class I gene.

Transgenic Line	Brain	Kidney	Spleen	Liver	Thymus
**PD1-CD2**	0	0	0	0	ND
**PD1-CD2/Bam(+)**	11.7	6.5	226.6	40.1	42.8
**PD1-CD2/Bam(−)**	1.0	13.6	206.6	12.1	55.3

RNA levels in each of the tissues were determined by qRT-PCR, normalized for 18S rRNA in the same sample.

ND – not determined. The results are the average of two independent experiments.

### Intronic sequences regulate tissue-specific MHC class I gene expression

To begin to map the minimal 3′ sequence requirements for stable MHC class I expression, we first truncated the 9 Kb genomic fragment used to generate the PD1 transgenic mouse, removing 3.7 Kb of distal intergenic sequences 3′ to the PD1 gene, leaving 730 bp immediately downstream of the polyadenylation site. In this construct (PD1/Bam), the 1 Kb regulatory region upstream of the TSS, the entire coding region and the poly A addition site remained intact (Figure 1A, second panel). All eight transgenic founder lines generated with this construct expressed class I on the surface of peripheral blood lymphocytes (PBL) (Figure 1A, second right panel). The overall pattern and level of cell surface expression in B- and T-cells (Figure 1B, compare top and bottom panels) and of RNA expression in tissues (Figure 1C; Table 2; Figure S1) of the PD1/Bam mice paralleled that previously reported for the original full-length 9 Kb MHC class I transgene and endogenous MHC class I genes [22]. Furthermore, PD1 transcription in PD1/Bam mice was activated by *in vivo* interferon treatment (Weissman, unpublished observations). Thus, all of the regulatory elements necessary for appropriate MHC class I gene expression *in vivo* reside within the 5.3 Kb PD1/Bam DNA segment.

**Table 2:** Summary of PD1 transgene expression in different lines of PD1 transgenic mice.

Transgene	Normal Expression in Iindependent Lines	Line	Expression by qRT-PCR					
			Animal	Spleen	Kidney	Liver	Brain	Thymus
PD1/Bam	8/8	N/A	N/A	12.7	2.3	2.9	1.6	10.9
PD1cDNAint1-2/Bam	8/8	418A4	#1	18.6	2.4	1.3	3.0	30.0
		418A4	#2	29.5	0.12	1.5	0.41	1.0
		418E4	#1	16.3	0.5	2.1	3.5	8.1
		418E4	#2	22.0	1.6	1.3	1.8	1.8
PD1cDNAint1-2/Sac	2/10	564I7 (expresser)	#1	10.7	1.0	1.2	2.5	9.5
		564I7 (expresser)	#2	20.5	1.5	0	3.3	14.0
		564K6 (non-expresser on PBL)	N/A	0	0	0	0	0
PD1cDNAint1-2/PolyA	7/21	730W6 (expresser)	N/A	7	0.06	0.6	0.5	0.7
		730N3 (non-expresser)	N/A	0	0.1	0	0	0
C57BL/6	N/A	N/A	N/A	0	0	0	0	0

RNA levels in each of the tissues were determined by qRT-PCR, normalized for 18S rRNA in the same sample.

To further map the necessary downstream elements and to investigate their role(s) in regulating expression, we generated transgenic mice from a series of constructs in which introns were successively removed. A transgenic construct deleted of introns 3–6 (Figure 1A) (PD1cDNAint1-2/Bam) directed normal levels (Figure 1A, third panel; Table 2) and patterns (Figure 1B, C; Table 2; Figure S1) of expression *in vivo*. A transgenic construct further deleted of all introns except intron 7 (PD1cDNA/Bam), was also expressed *in vivo* (Figure 1D), as assessed by the presence of transcripts in PBL and tissues. However, in contrast to the PD1cDNAint1-2/Bam construct containing introns 1 and 2, expression was barely detectable in PBL (Figure 1A, bottom panel) and highly aberrant in other tissues relative to the genomic transgene: Expression was disproportionately high in brain and low in spleen, relative to liver, as assessed by RT-PCR or by northern (Figure 1D).

Taken together, these data demonstrate that while PD1 promoter activity *per se* does not depend on the introns, introns 1 and/or 2 contain sequences necessary for normal tissue-specific patterns of PD1 expression *in vivo*. Importantly, the reproducibility of PD1cDNAint1-2/Bam expression and proper tissue distribution among multiple independent transgenic lines indicates that this short 4.0 Kb construct contains the minimal PD1 regulatory unit.

### 3′ intergenic sequences are necessary for MHC class I expression *in vivo*

**Figure 2:**
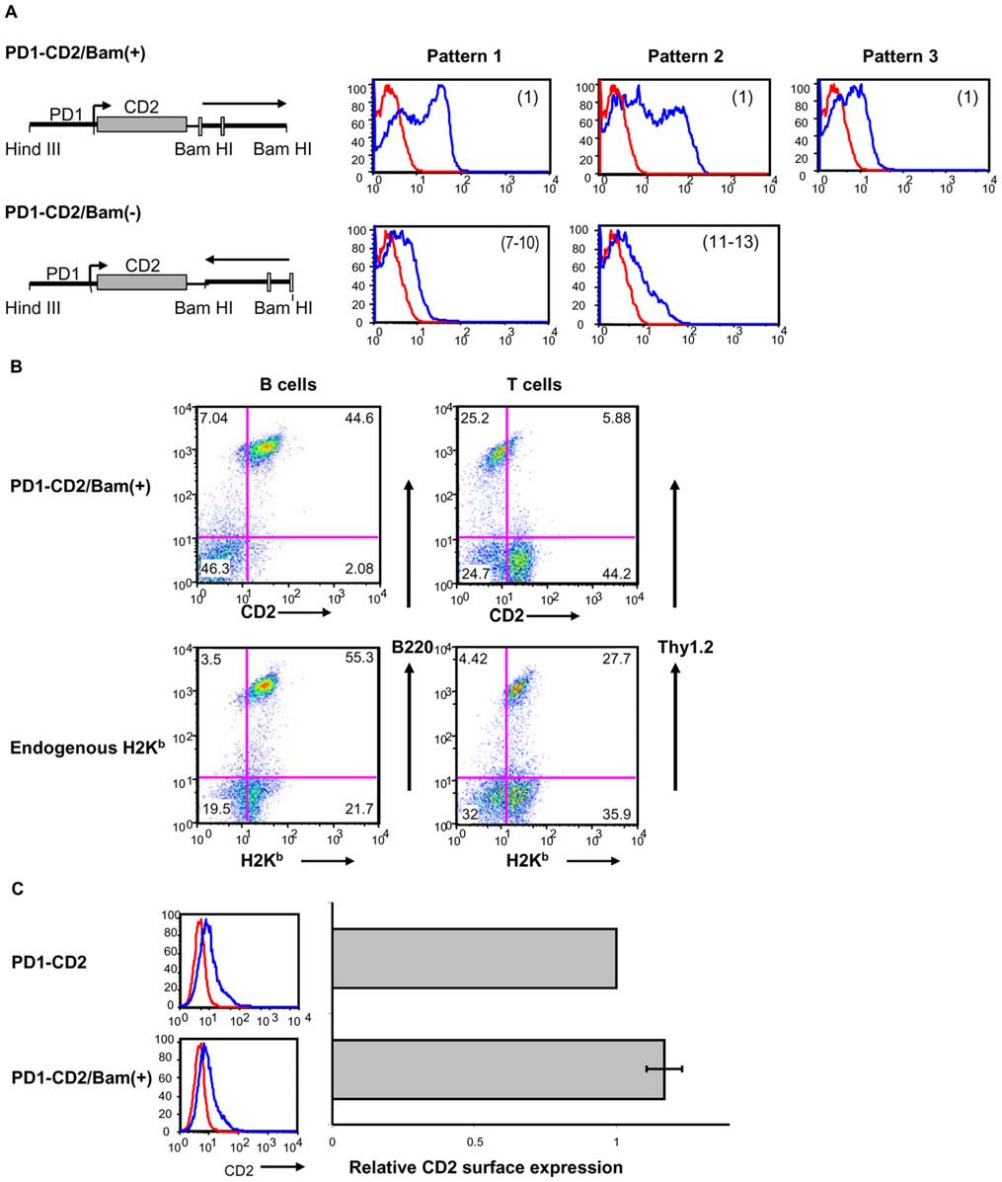
3′ intergenic sequences are required for stable *in-vivo* expression of the MHC I gene, PD1. (A) FACS profiles of CD2 surface expression on PBL of transgenic mice. Transgenic mice were generated with constructs containing the human CD2 reporter driven by the PD1 promoter (PD1/CD2) into which a segment 3′ to PD1 coding sequences was inserted in either the forward (PD1-CD2/Bam(+)) or reverse orientation (PD1-CD2/Bam(−)). The numbers in parentheses indicate the range of transgene copy numbers in different lines. (B) FACS profiles of huCD2 expression on B (B220) and T (Thy1.2) cells of PD1CD2/Bam(+) transgenic mice (pattern 1 from (A)). H-2K^b^ expression pattern in the same mice is shown as control. (C) M12 cells were transiently transfected with PD1/CD2 or PD1-CD2/Bam(+). Surface expression was determined by FACS analysis with anti-huCD2 antibody (left panels); relative surface expression (right panels) is the average level of expression and standard deviation among 3 independent transfections.

The finding that *in vivo* expression of the PD1 gene does not depend on introns 1–6 suggested that the required downstream sequences are located within the 3′ intergenic region, which is present in all of the constructs that are expressed *in vivo*. To test this possibility, we next asked whether the 3′ terminus of the PD1/Bam clone could restore expression to the silent PD1-CD2 transgene (Figure 1A). To this end, a 1.3 Kb BamHI fragment encompassing the poly A addition site and 730 bp of 3′ intergenic sequences was inserted, in both orientations, downstream of the CD2 coding sequence in the PD1-CD2 construct (PD1-CD2/Bam(+) or PD1-CD2/Bam(−); Figure 2A, upper and lower panels, respectively). In contrast to the parental PD1-CD2 construct, all of the transgenic lines containing the PD1-CD2/Bam(+) construct (5/5) and a majority (7/8) of those with the PD1-CD2/Bam(−) expressed CD2, both on their PBL and in their tissues. (Figure 2A; Table 1). Those with the 3′ Bam segment in the forward orientation generally expressed cell surface CD2 protein at higher levels on PBL, despite their lower copy number (Figure 2A). Thus sequences downstream of the PD1 coding region contain regulatory elements necessary for expression.

Although the PD1-CD2/Bam(+) mice expressed surface CD2 on PBL, the patterns of expression of the CD2 transgene on lymphocyte populations and in the tissues differed markedly from the endogenous MHC class I and PD1 transgene patterns (Figure 2B; data not shown). These results are consistent with the observation that introns 1 and/or 2 of the PD1 gene are necessary for proper tissue expression.

The 3′ regulatory element(s) necessary for *in vivo* expression could function either as enhancers that augment promoter activity or as barrier elements that prevent silencing by encroachment of heterochromatin. However, the 3′ segment does not function as a classical enhancer since the presence of the 1.3 Kb BamHI 3′ segment in PD1-CD2/Bam(+) did not significantly augment class I promoter activity relative to PD1-CD2 in transient transfections of either murine L cells or human HeLa cells (Figure 2C).

These findings indicate the presence of a barrier element in the 3′ intergenic segment of the PD1 gene.

### Sequence elements 3′ to the MHC class I gene have barrier function

**Figure 3:**
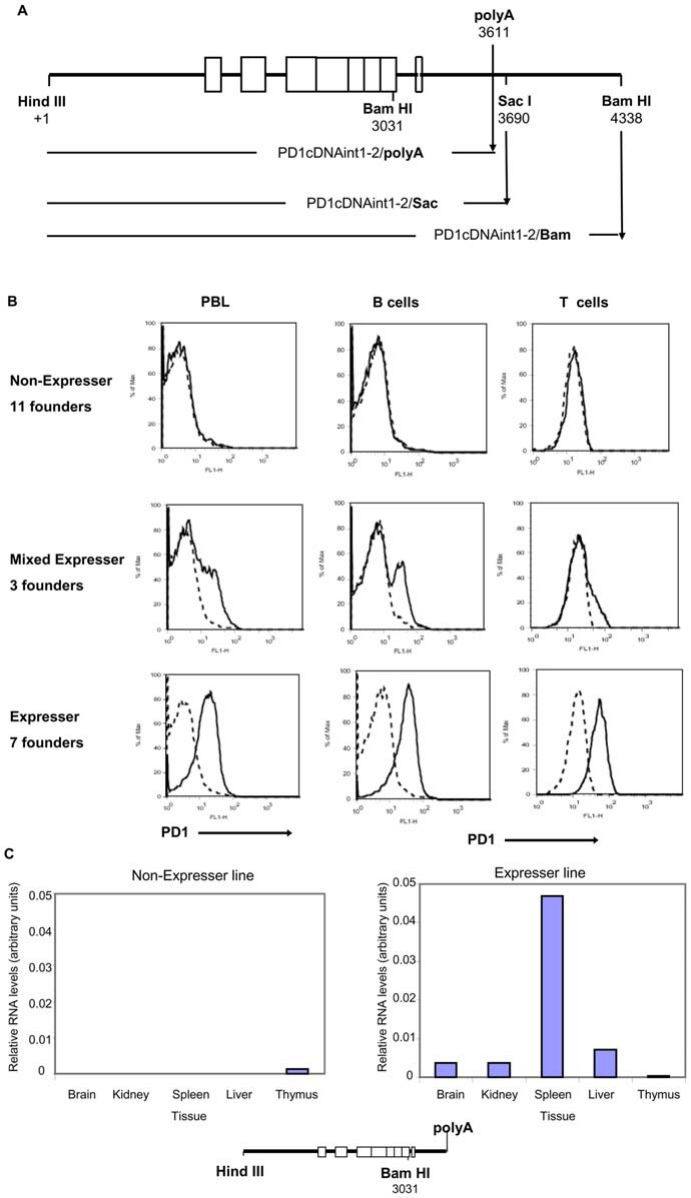
Expression of a PD1 transgene is variegated in the absence of sequences 3′ to the polyA site. (A) The positions of the polyA, SacI and BamHI sites in the PD1cDNAint1-2/Bam construct are diagrammed and constructs generated from truncation at these sites indicated. Numbers indicate the position relative to Hind III site at +1. (B)PD1 expression is variegated on PBL among transgenic lines carrying a PD1cDNAint1-2 construct truncated at the polyA site. PBL from the different PD1cDNAint1-2/polyA transgenic lines were stained with either anti-PD1 antibody alone (left panels) or in combination with anti-B220 (B cells) (middle panels) or anti-Thy1 (T cells) (right panels). Shown are representative PD1 profiles of each of the groups. The map of the construct is shown at the bottom. (C) RNA levels in different tissues of non-expresser and expresser PD1cDNAint1-2/polyA transgenic lines were assessed by qPCR. Results shown from individual mice are representative of 2–3 individuals tested for each line. (The small amounts of RNA detected in the thymus are not reproducibly observed.)

The minimal 3′ sequences necessary for barrier activity were determined by 3′ truncation of PD1 cDNAint1-2/Bam intergenic sequence to the poly A addition site (PD1 cDNAint1-2/PolyA) (Figure 3A). Most of the transgenic founders generated with this construct (14/21) either failed to express in any tissue or displayed a variegated pattern of expression on PBL (Figure 3B, C, left panels). Only 7 of the founders displayed low level PD1 expression on the surface of PBL and a normal pattern of RNA expression in B and T cells and in the tissues (Figure 3B right panels, Figure 3C, Table 2).

This variegation in the expression of the PD1cDNAint1-2/PolyA construct among the independent founder lines contrasts sharply with transgenic mice derived with the PD1cDNAint1-2/Bam, all eight of which expressed with normal tissue-specific patterns. Therefore, sequences within the 3′ intergenic region are required for normal *in vivo* expression of PD1 and thus provide barrier function.

**Figure 4:**
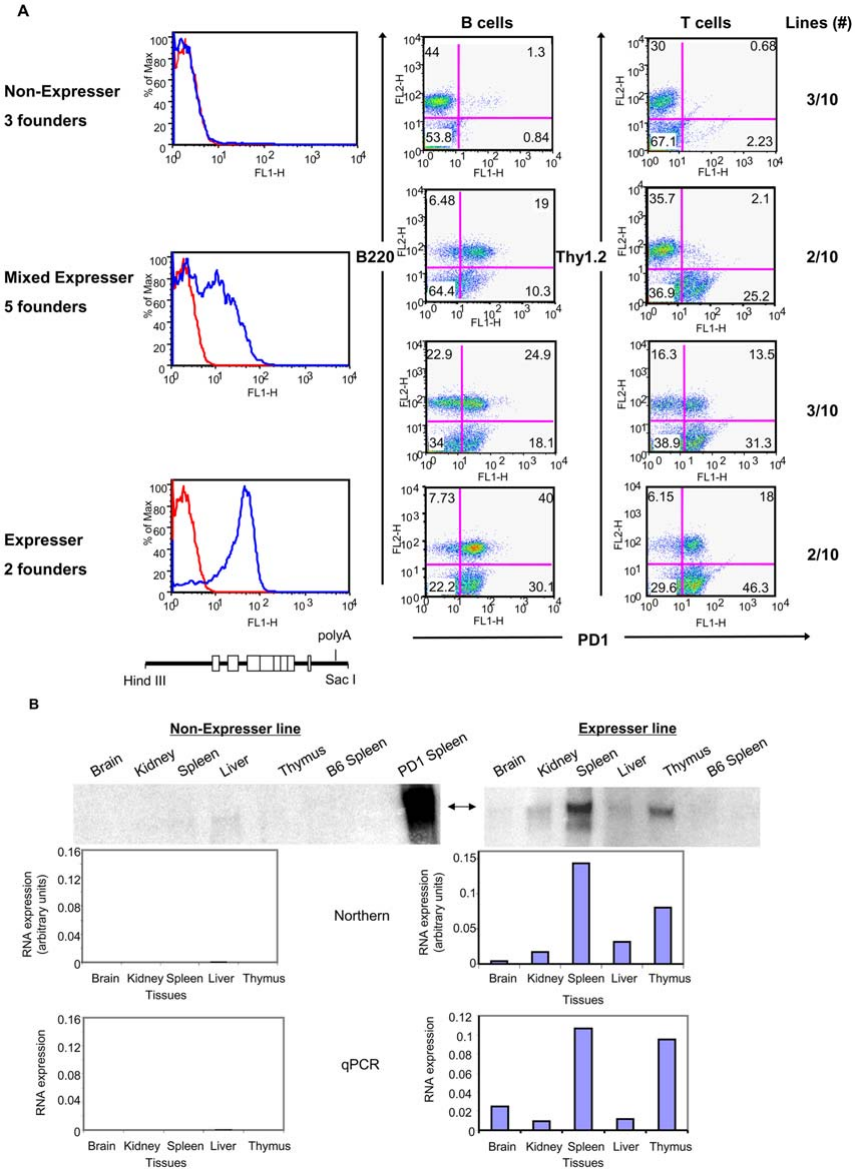
Expression of a PD1 transgene is variegated in the absence of sequences 3′ to the SacI site. (A) PD1 expression is variegated on PBL among independent transgenic founder lines carrying a PD1cDNAint1-2 construct truncated at the SacI site. PBL from the different PD1cDNAint1-2/Sac transgenic lines were stained with either anti-PD1 antibody alone (left panels) or in combination with anti-B220 (B cells) (middle panels) or anti-Thy1 (T cells) (right panels). Shown are representative profiles of each of the groups. Numbers at the right show the number of founder lines having the same pattern of expression. The map of the construct is shown at the bottom. (B) RNA levels among the different tissues of non-expresser (left panels) and expresser (right panels) PD1cDNAint1-2/Sac transgenic lines were assessed by Northern using a probe that spans exons 2–3 and only minimally cross-reacts with endogenous MHC class I (top panels); level of expression was quantitated relative to 18S RNA (middle panels). RNA levels in various tissues of an independent set of mice were also measured in by qPCR and quantitated relative to 18S RNA (bottom panels). Results shown from individual mice are representative of 2–3 individuals tested for each line.

Similar variegated expression was observed when the PD1 cDNAint1-2/PolyA, which does not retain barrier activity, was extended by 79 bp to a downstream Sac site (PD1 cDNAint1-2/Sac; Figure 3A, 4A). Of 10 founder lines generated from this construct, only two showed levels of cell surface expression on PBL comparable to the PD1cDNAint1-2/Bam mice, with a distribution between T and B cells that paralleled PD1cDNAint1-2/Bam transgenic lines (Figure 4A, compare with Figure 1B). The remaining lines either did not express PD1 (3/10) or had aberrant expression patterns (5/10) on whole PBL, T cells and B cells (Figure 4A).

Variegated patterns of RNA expression among the lines were observed in the tissues as well, as determined by quantitative RT real-time PCR of PD1 transcripts and Northern analysis (Figure 4B, Table 2). Thus, restoration of only the 79 bp immediately downstream of the PolyA addition site is insufficient to overcome variegated expression. These results suggest that stable transgene expression depends, at least in part, on regulatory element(s) located in the 650 bp segment between the Sac I site and the distal Bam H1 site (see Figure 3A).

### 3′ intergenic sequences are necessary to maintain MHC class I expression in stably transfected cell lines

**Figure 5:**
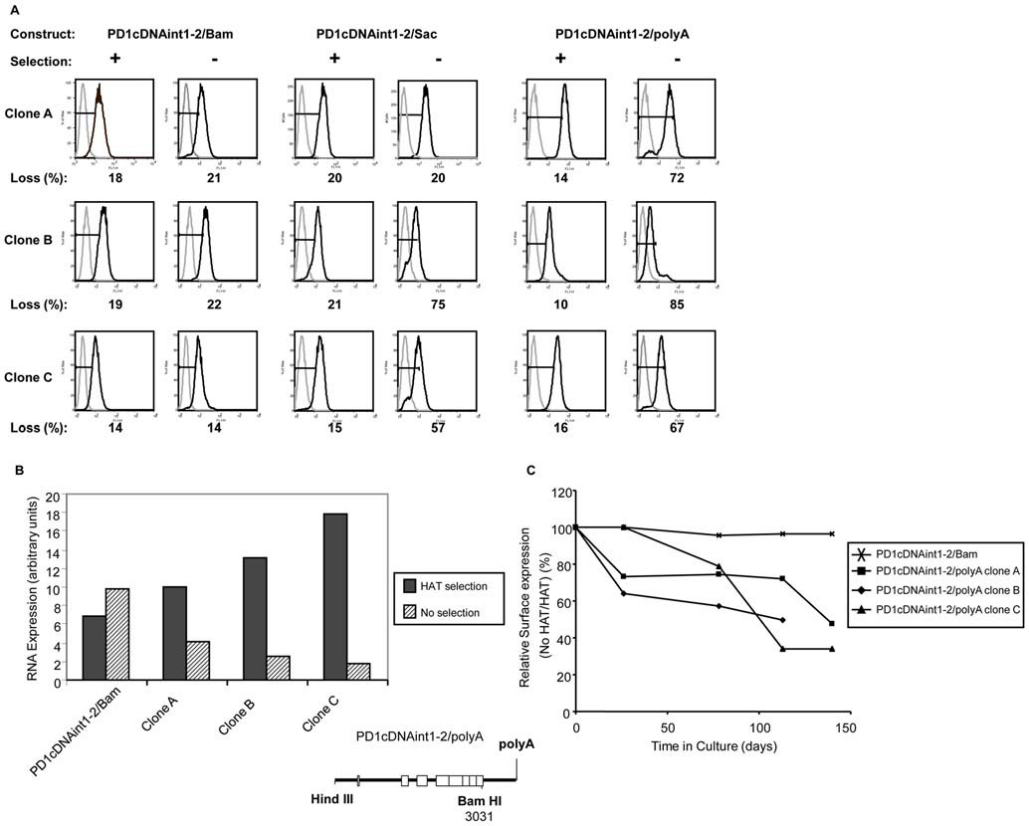
Sequences 3′ to the MHC I gene are required to maintain expression in stably transfected cells. (A) FACS profiles of three representative stable transfectant L cell clones (A,B,C) derived from PD1cDNAint1-2/Bam, PD1cDNAint1-2/Sac and PD1cDNAint1-2/polyA maintained with or without selection for 100 days and stained with anti-PD1 antibody. Gray curves represent the negative control. The numbers below the profiles are the percentage of cells that have lost PD1 expression. Copy numbers in all lines varied between 2 and 6. (B) Relative PD1 RNA levels in PD1cDNAint1-2/Bam and PD1cDNAint1-2/polyA clones A–C maintained with or without selection for 100 days, as determined by RT qPCR. Clones A–C correspond to the respective clones in (A). (C) Kinetics of loss of PD1 surface expression of PD1cDNAint1-2/polyA lines after transfer to non-selective medium. Clones grown in the presence and absence of HAT for various times were assessed for surface PD1 expression by staining with anti-PD1 antibody and FACS analysis. The relative level of surface expression is defined as the ratio of mean fluorescence units of the clones grown in the absence of HAT relative to the same clone grown in the presence of HAT.

To further characterize the barrier activity of the 3′ intergenic sequences, we turned to an *ex vivo* assay. Previous studies in cultured cell lines have demonstrated that expression of stably transfected genes lacking barrier elements is gradually extinguished in the absence of selective pressure, as heterochromatin propagates into the transgene [30]. Therefore, we examined whether 3′ intergenic sequences are required to maintain PD1 expression in stably transfected cells, as follows. Stable L cell transfectant clones of PD1cDNAint1-2/Bam, PD1cDNAint1-2/Sac or PD1cDNAint1-2/PolyA constructs were isolated and maintained in parallel without or with selective HAT medium. Boundary function of the 3′ intergenic region was assessed by periodic monitoring of PD1 expression. Clones generated with any of the three constructs all expressed comparable levels of cell surface MHC class I when maintained in HAT selection (Figure 5A). Clones transfected with PD1cDNAint1-2/Bam all maintained expression for at least 4 months in the absence of HAT (Figure 5A, left set of panels). In contrast, among clones transfected with constructs deleted to either the PolyA site (PD1cDNAint1-2/polyA) or to the SacI site (PD1cDNAint1-2/Sac), the majority of the clones lost expression in the absence of selection, as assessed both by surface expression and quantitative real-time RT PCR (representative patterns are shown in Fig. 5A, and Figure 5B; Table S2; the time course of loss of expression of three of these clones is shown in Figure 5C). Thus, variegated loss of expression among the stably transfected L cell clones paralleled their expression pattern in transgenic mice, further establishing the function of the 3′ intergenic region as a barrier element.

**Figure 6:**
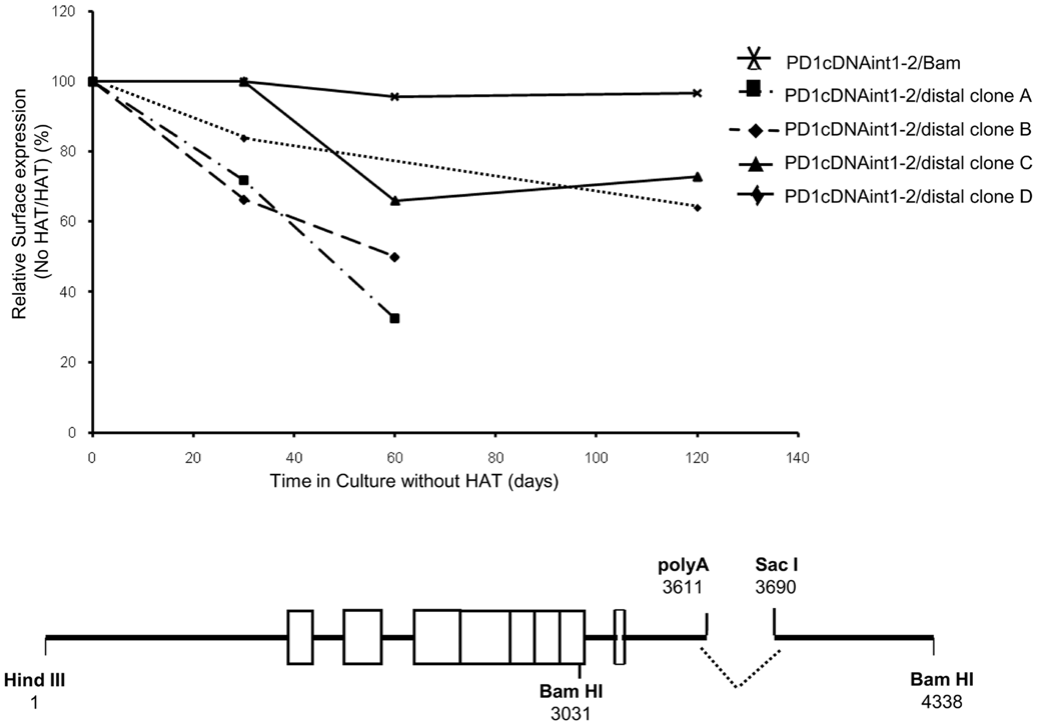
Loss of PD1 expression following deletion of 79 bases within the 3′ intergenic region. Kinetics of loss of PD1 surface expression from L cell lines stably transfected with PD1cDNAint1-2/distal after transfer to non-selective medium. Expression was monitored as described in Figure 5. The PD1cDNAint1/2 construct from which 79 base pairs, between the polyA and SacI site were replaced with pUC19 sequences, to generate PD1cDNAint1-2/distal is illustrated at the bottom.

We used this *ex vivo* barrier assay to further define the barrier element. Since the intergenic 79 bp segment between the polyA site and the Sac I site alone did not restore barrier function either *in vivo* or in L cell clones, we next asked whether the presence of the distal intergenic segment between the Sac I site and the Bam HI site alone was sufficient to restore barrier function. To this end, the 79 bp polyA-SacI segment within the stably expressed PD1cDNAint1-2/Bam was replaced by an irrelevant pUC19 DNA sequence (PD1cDNAint1-2/Distal; Figure 6, bottom). Of nine stable L cell clones transfected with this construct, seven lost expression to varying degrees in the absence of selection (Figure 6 shows the time course of loss of expression for four of the clones; Table S3). Thus, the distal 650 bp segment between the Sac and Bam sites is not sufficient to maintain stable expression.

Since neither this segment nor the 79 bp interval between the Poly A and Sac sites are able to provide complete barrier function independently, both segments contribute to full barrier function.

If the 3′ intergenic segment downstream of the polyA addition site contains barrier function, as indicated by the above data, we would predict that it would not be required for promoter activity in the context of a minichromosome that is not integrated into genomic DNA. Indeed, when cloned into the bovine papilloma viral vector, p-REP7, and introduced into the human K562 cell line (where it is maintained as an extrachromosomal episome packaged into nucleosomes but not subject to heterochromatin encroachment), the PD1cDNAint1-2/polyA construct is stably expressed, both in the presence and absence of the selective medium (data not shown).

Taken together, these studies functionally identified a complex boundary element within the 3′ downstream sequence of the MHC class I gene between the poly A addition site and the distal Bam site. Full boundary function depends on the presence of both the 79 bp segment downstream of the polyA site and the DNA segment between the Sac I site and the distal Bam H1 site (see Figure 3A). Neither DNA segment alone is sufficient to confer barrier activity; both are necessary. Whether barrier activity is mediated by a continuous DNA element spanning the SacI site, or by two discontinuous segments, remains to be determined.

### Element(s) within the 3′intergenic region are necessary to maintain active MHC class I chromatin organization and modifications

**Figure 7:**
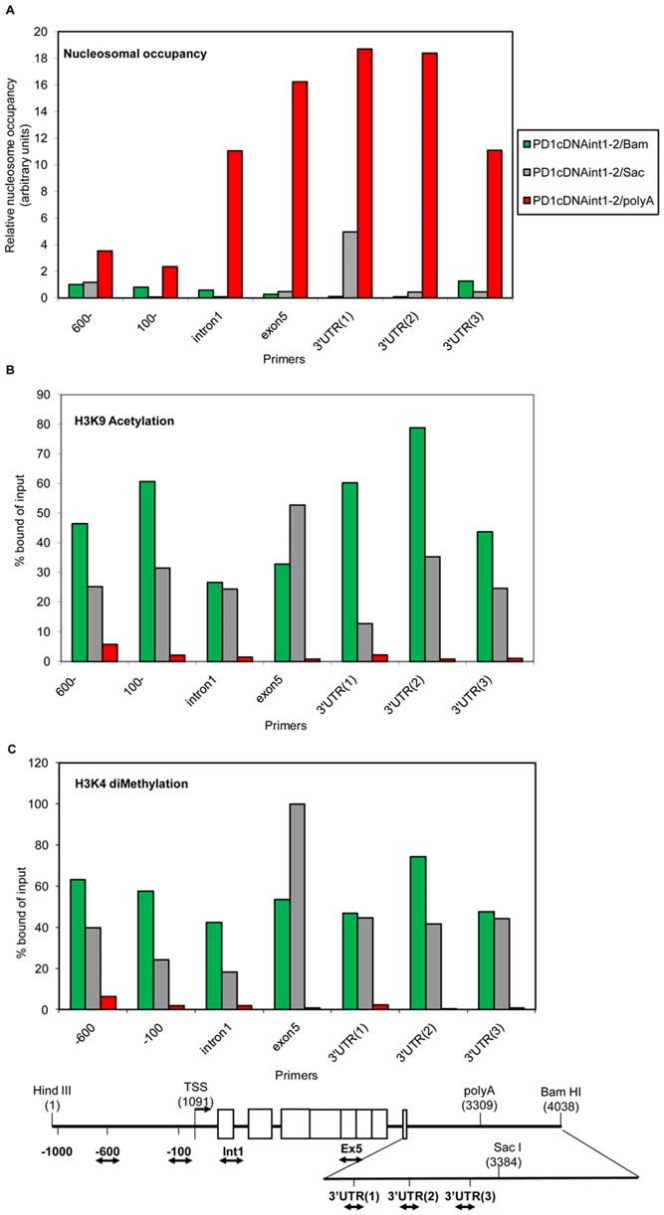
Altered chromatin organization is associated with deletions in the 3′ intergenic region, independent of expression. (A)The relative nucleosome density across the PD1 gene was determined as described in Materials and Methods in L cells stably transfected with PD1cDNAint1-2/Bam, PD1cDNAint1-2/Sac and PD1cDNAint1-2/polyA and maintained in the absence of HAT medium. Although all three clones retain the respective transgenes, only the PD1cDNAint1-2/Bam line expresses PD1. The results shown were derived with clones B in Figure 5A. The location of primers used for determining nucleosomal occupancy are illustrated in the map below the graph. The abscissa corresponds to the sites amplified as indicated on the map. (B) Relative levels of histone H3 K9/14 acetylation, and (C) histone H3K4 dimethylation. Materials and Methods, primers and abscissa are the same as in (A). All results are representative of two to three ChIP of independent clones.

Barrier elements are required to maintain nucleosome organization and histone modifications associated with an open chromatin structure [30]. Thus, the presence of barrier activity in the 3′ intergenic region downstream of the polyA site predicts that both the nucleosome organization and histone modifications of the class I gene will be affected by its absence. To determine whether chromatin changes accompany gene silencing, we assessed the effect of the barrier segment on the relative nucleosome density across the gene in stable L cell transfectant clones. In the selective medium, where expression is maintained for all constructs, all three showed similar pattern of uniformly low nucleosomal density (Figure S3). In the absence of selection, nucleosomal density remained uniformly low across the PD1 gene in PD1cDNAint1-2/Bam (which stably expressed PD1). In sharp contrast, PD1cDNAint1-2/polyA clones that lost expression acquired significantly increased nucleosomal density across the gene (Figure 7A). Thus, nucleosomal density across the class I gene correlates with expression status and with the presence of the full 730 bp intergenic segment containing 3′ barrier function.

Surprisingly, loss of expression of the PD1cDNAint1-2/Sac construct does not correlate with nucleosomal density, which was almost uniformly low across the gene and indistinguishable from the expressing PD1cDNAint1-2/Bam clone (Figure 7A). These findings indicate that whereas the entire region extending from the Poly A site to the distal Bam site is necessary for stable expression, the activity located in the 79 bp between polyA and the Sac I site is sufficient to maintain an “open” chromatin structure. Thus, there are two separable barrier element activities.

To examine the relationship between barrier function and histone modifications, we assessed chromatin modifications associated with active chromatin across the gene in the same clones that were analyzed for nucleosomal density. Specifically, we assayed by ChIP analysis for the presence of histone H3K9/K14 acetylation and H3K4 dimethylation. As shown in Figures 7B and C, histone H3K9/K14 acetylation and H3K4 dimethylation modifications were reduced along the non-expressing PD1cDNAint1-2/polyA construct compared to the PD1cDNAint1-2/Bam construct, in agreement with the increase in nucleosomal abundance. Surprisingly, but consistent with its low nucleosomal abundance, the non-expressing PD1cDNAint1-2/Sac transfected clones displayed histone modifications across the gene at levels comparable to the fully-expressing PD1cDNAint1-2/Bam transgene clones. Thus, the 79 bp between the PolyA addition site and the Sac site contribute to both barrier function and maintenance of histone modifications associated with an active chromatin conformation. Importantly, however, active chromatin organization is not sufficient for stable expression.

Interestingly, we did not observe increases in modifications associated with stable heterochromatin, namely DNA CpG methylation (Figure S2) or histone H3K9 di-methylation (data not shown), on the promoter of the PD1cDNAint1-2/polyA.

### CBP,PCAF and H2A variant H2A.Z are associated with the MHC class I gene barrier region

**Figure 8:**
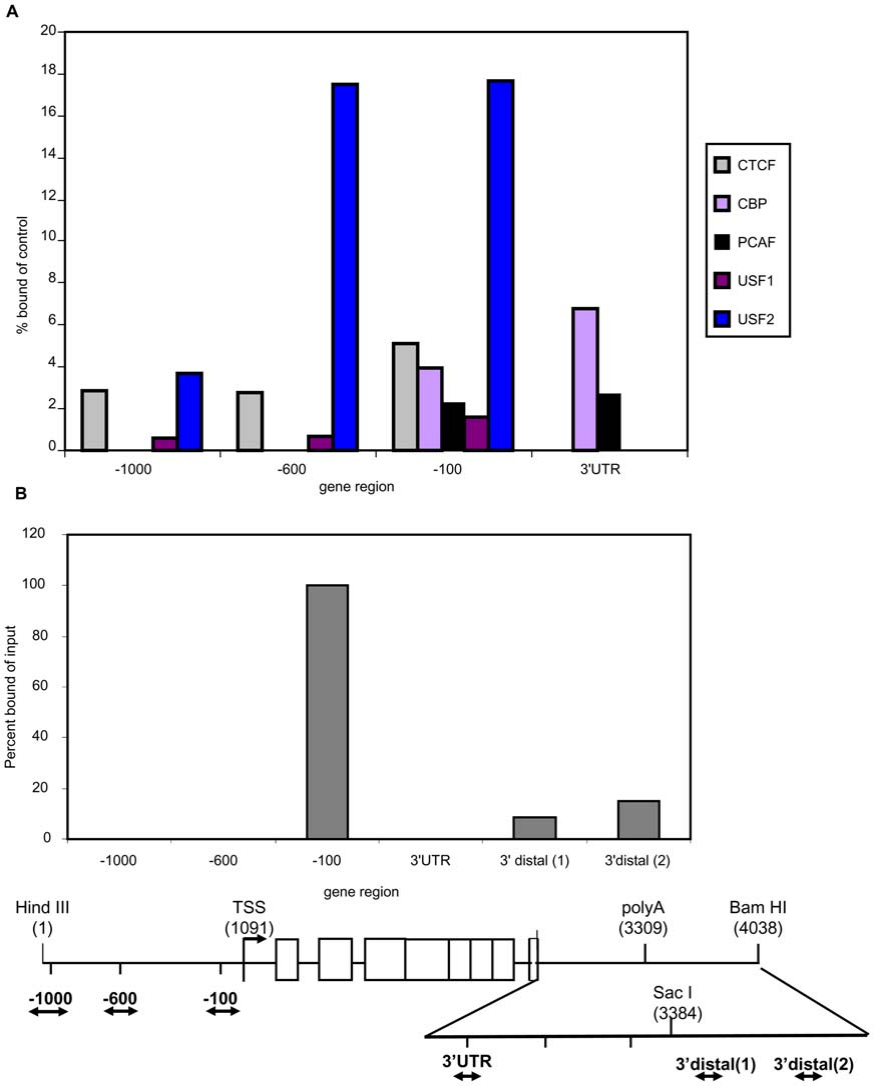
Histone modifying enzymes and histone variant H2A.Z are associated with PD1 3′ intergenic sequences. (A) ChIP analysis was performed on L cells stably transfected with PD1cDNAint1-2/Bam, as described in Materials and Methods, to determine the extent of association of CTCF, CPB, PCAF, USF1 and USF2 across the PD1 gene, as indicated. All three 3′ UTR primers gave the same results; only 3′UTR(1) is shown. (B) The binding of histone variant H2A.Z across the PD1 gene was similarly determined in the same clone. All results shown are representative of two ChIP experiments of independent clones. None of the 3′UTR primers showed any amplification following ChIP with anti-H2A.Z.

Histone modifying enzymes have been reported to be associated with barrier elements, thereby actively regulating chromatin modifications [31]. In light of the differences in chromatin organization and modifications among the constructs, we assayed by ChIP for the presence of the histone acetyl transferase enzymes, CBP and PCAF across the PD1cDNAint1-2/Bam gene in L cells. Indeed, we found that both CBP and PCAF were associated with the 3′ intergenic region as well as with the promoter (Figure 8A).

Among the transcription factors that have been identified within boundary complexes are Upstream Factors 1 and 2 (USF1 and USF2), which recruit histone modifying enzymes to the beta-globin barrier element [31], [32] and occupy the alpha-spectrin barrier element as well [33]. Although both factors were found at upstream sites, associated with the extended promoter (Figure 8A; [34]), neither factor was detected in the class I 3′ boundary region, despite the presence of multiple E box sequences within the 3′ intergenic region (Figure 8A; Table S1). These results indicate that a different mechanism is employed for the recruitment of the above enzymes to the MHC class I barrier element than has been reported for the chicken beta globin boundary element.

The enhancer-blocking factor, CTCF, interacts with both β-globin [35] and MHC class II [36] boundary elements which contain both barrier and enhancer blocking functions. However, we found no evidence in PD1cDNAint1-2/Bam in L cells that CTCF interacts with the MHC class I barrier element (Figure 8A). In contrast, CTCF was clearly detected within 600 bp of the PD1 promoter (Figure 8A).

The presence of histone variant H2A.Z in chromatin has been associated with barrier function [37]–[39] and with promoters [40]–[42]. Indeed, H2A.Z was associated both with sequences in the 3′ barrier region of PD1cDNAint1-2/Bam and at higher levels at the 5′ promoter region (Figure 8B)

In summary, the above studies have demonstrated the presence of a complex barrier activity within the 3′ intergenic region of the MHC class I, PD1, gene that is required for stable expression of the gene and for maintaining an open chromatin structure.

## Discussion

The MHC class I genes, whose products play a critical role in immune homeostasis, are primarily regulated transcriptionally. Past studies on understanding the relationship between transcriptional regulation and immune function have largely focused on characterizing the regulatory elements and transcription factors that govern their transcription. The core promoter and 5′ extended promoter region of the MHC class I gene, PD1, contain elements that mediate regulation of both constitutive, tissue-specific levels of MHC class I expression and of immune activated expression [2], [3], [5], [6], [24], [25]. We now have extended the identification and characterization of critical transcriptional regulatory elements to sequences downstream of the promoter. We find that normal expression of the MHC class I gene, PD1, *in vivo* depends on both intronic and 3′ intergenic sequences. Element(s) within the introns are necessary for normal, tissue-specific levels of expression. A novel barrier element located in the 3′ intergenic region is necessary for sustained *in vivo* PD1 promoter activity and thus is an important component of MHC class I immune function.

Although transcribed at very different levels in different tissues, the PD1 promoter is constitutively in an open chromatin conformation regardless of transcriptional activity, enabling the cells to respond rapidly to infection by intracellular pathogens by increasing MHC class I transcription rates without chromatin remodeling [28]. In the current study, we show that this open chromatin conformation depends on the presence of the 3′ intergenic barrier element, which blocks heterochromatin encroachment and silencing. Removal of the barrier element results in loss of expression accompanied by increased nucleosomal occupancy and reduced active chromatin modifications across the gene. Thus, the barrier element has two functions: (1) maintaining stable expression, and (2) maintaining active chromatin modifications. Importantly, these two functions are distinct and physically separable: partial truncation of the barrier element results in loss of expression without a concomitant loss of the active chromatin modifications. These findings lead to the conclusion that an active chromatin structure is not sufficient to support gene expression, absent other elements within the barrier.

The separation of chromatin modifying activities and sustained expression found in the mammalian MHC class I, PD1, barrier element is similar to that observed in the chicken β-globin 5′ compound boundary element that separates the gene locus from an upstream 16 Kb heterochromatin domain [31]. This boundary element consists of five protein binding sites, all of which are necessary for its function. Recruitment of histone modifying enzymes to one of these sites terminates heterochromatin spreading by generating an active chromatin conformation at the barrier. Similar to the PD1 barrier, recruitment of histone modifying enzymes and the resulting histone modifications at the β-globin promoter and gene are necessary but not sufficient for the barrier activity [31]. We speculate that in both cases genomic barriers require the combined function of distinct complexes with different activities.

Although alone it is not sufficient to sustain transcription, active chromatin structure contributes to MHC class I barrier function. The binding of CBP and PCAF to the 3′ intergenic region of PD1 suggests that the mechanism of the MHC class I barrier function involves active recruitment of histone modifying enzymes. Indeed, histone modifying enzymes have been implicated in mediating the activities of barriers associated with other genes [31], [32], [43], [44]. We speculate that CBP and PCAF may be involved in the anti-silencing function of the MHC class I barrier element. The precise sequences that are involved in recruitment of histone modifying enzymes are yet to be determined. It is possible that they are within or flanking the 79 bp sequence downstream of the polyadenylation signal, deletion of which resulted in altered chromatin arrangement and loss of expression.

Since neither CBP nor PCAF bind DNA directly, they must be recruited to the barrier element by one or more transcription factors. USF1/2 has been shown to play a critical role in mediating barrier activity of the chicken β globin gene, recruiting histone modifying enzymes [31], [32]. USF1/2 is also associated with a barrier element located within the alpha-spectrin gene [33]. However, no binding of USF1 or USF2 to the PD1 3′ intergenic region was detected, either *in vivo* or in *in vitro* gel shift assays (data not shown), despite the presence of a consensus E box binding sites within the 3′ intergenic barrier region. The transcription factor(s) that recruits CBP and PCAF to the PD1 barrier remains to be identified. In initial gel shift studies with HeLa nuclear extract, a complex dependent on sequences within the 79 bp segment between the polyA addition site and the Sac I site was observed (data not shown). However, the sequence was not homologous to any known canonical binding sites and it was not possible to identify the cognate factors. The HMG box containing protein, HBP1, has been shown to bind to an element with barrier function within the locus control region of the human CD2 gene [45]. Although a half-site occurs with the PD1 3′ intergenic sequence, the full HBP1 binding site, TTCATTCATTCA, does not. Moreover, DNA sequence homology searches did not identify any additional known binding sites within the entire 3′ intergenic region.

The histone variant, H2A.Z, interacts with both promoters and barrier elements in yeast [37]–[39] and vertebrates [40], [41], suggesting that it also may contribute to barrier function. The association of H2A.Z with the intergenic region suggests it may take part in the barrier function of the PD1 gene as well.

Interestingly, loss of PD1 expression did not depend on the acquisition of repressive chromatin marks. Modifications associated with silenced genes, H3K9 dimethylation and promoter CpG methylation, did not appear in the absence of the barrier element. Consistent with these findings, several studies have shown that transcriptional silencing by heterochromatin propagation is a gradual process, composed of stepwise formation of heterochromatin, leading to the existence of intermediate state heterochromatin, in which silencing is partial and histone modifications are a mixture of active and inactive chromatin [46]–[48] while DNA CpG methylation appears long after the gene is silenced [48]. The latter modification appears to be involved in maintenance, rather than establishment, of transcriptional silencing [48], [49]. In accordance with these observations, lack of H3K9 dimethylation and DNA methylation of the PD1 transgene truncated at the barrier site is consistent with intermediate transcriptional silencing and suggest the existence of yet other elements that contribute to boundary activity.

In conclusion, the PD1 barrier element, like other functionally defined barrier elements, serves to sustain gene expression and to maintain histone modifications and low nucleosomal density associated with active transcription [30]–[32], [38], [44], [48], [50]–[63]. In the complete absence of the intergenic barrier element, expression is extinguished with the accompanying loss of histone modifications and increased nucleosomal density. Surprisingly these modifications are not sufficient to retain expression. Loss of both expression and chromatin modification requires truncation of the entire intergenic segment. The striking conclusion from these findings is that active chromatin modifications and structure are not sufficient to maintain expression of the MHC class I, PD1, gene. Future experiments will further define the function of the MHC class I barrier element.

## Materials and Methods

### DNA Constructs

PD1/Bam extends from the Hind III site approximately 1 Kb 5′ of the PD1 promoter through the entire coding sequence (CDS),and 730 bp beyond the poly A addition sites to a Bam HI site in the 3′ intergenic region cloned in pBR322 (Figure 1A). PD1cDNAint1-2/Bam was generated from PD1/Bam by replacing a BbvcI/BamHI genomic fragment, spanning exon 3 through exon 7 with a BbvcI/BamHI cDNA fragment that contained only the exons (Figure 1A). PD1cDNAint1-2/Sac was generated by truncation of the PD1cDNAint1-2/Bam to the 3′ Sac I site 79 bp beyond the poly adenylation signal (Figure 3A). PD1cDNAint1-2/polyA was generated by cloning of the PD1 cDNA sequence into the Sal I site of pUC19 and replacing the 5′ segment containing the first two exons with a genomic HindIII/NcoI sequence including the 1 Kb upstream regulatory region through exon 3 thereby including the first 2 introns (figure 3A). To construct the PD1cDNAint1-2/distal clone, a DNA segment extending from the distal 3′ Sac I site to the distal BamHI site was inserted downstream of PD1cDNAint1-2/polyA (Figure 3A), into the BamHI site of the pUC19 vector, thereby replacing 79 bp PD1 with pUC19 sequences. The PD1/CD2 construct was generated by cloning a 1.1 Kb fragment containing the PD1 promoter upstream of the hCD2Δ74 cDNA construct (gift of D. Littman, NYU) (Figure 1A). PD1/CD2-3′(+) and PD1/CD2-3′(−) were generated from PD1/CD2 by inserting a genomic 1.3 Kb BamHI fragment of PD1, spanning intron 7, exon 8, the 3′UTR and 3′ intergenic sequences into the Sal/BstI site immediately 3′ of the CD2 gene (Figure 2A).

### Transgenic mice

Transgenic mice were generated as described previously [22], and screened by either Southern blotting or PCR of genomic tail DNA. All animal work was approved by the NIH Animal Care and Use Committee. Animals were housed and handled in strict accordance with ALAC requirements under the NIH protocol EIB-086.

### Transfections

For transient transfection assays, 1×10^6^ HeLa cells were transfected with 10 µg of DNA using Lipofectamine (Gibco/Invitrogen, Grand Island, NY) according to manufacturer's protocol. M12 cells were transfected as described before [3].

For stably transfected Ltk(−) clones, 1×10^6^ cells were co-transfected with 0.5 µg of the indicated clone DNA and 10 µg plasmid containing the thymidine kinase gene (TK) using Lipofectamine. The cells were maintained in DMEM for 48 hours and then the medium was replaced with fresh DMEM containing 1% hypoxanthine-aminopterine-thymidine (HAT). Isolated clones were analyzed for PD1 expression by FACS.

### Flow cytometry

FACS was carried out as described before [64], using antibodies detailed below; except for PD1 surface expression, in which case cells were stained with unlabeled primary Ab followed by goat anti-mouse secondary Ab conjugated to FITC (SouthernBiotech, Birmingham, AL). FACS results were analyzed using FlowJo. Antibodies: PD1 surface expression in mouse peripheral blood lymphocytes (PBL) and mouse cell lines was analyzed using PT85 anti-SLA antibody (VMRD Inc, Pullman, WA) while human cell lines were screened using the anti-SLA antibody 74-11-10 (VMRD). Other antibodies used for FACS analyses were: human CD2 (Pharmingen), B220 (BD-Pharmingen), Thy1.2 (BD-Pharmingen), H-2 Kb (BD-Pharmingen).

### Northern blot analysis and real-time RT-PCR

RNA was prepared using Trizol (Invitrogen). Northern blotting and real-time RT-PCR for PD1 analyses were done as described before [28]. For CD2 blots, full-length human CD2 cDNA fragment was used to probe for CD2 RNA.

### Nucleosome density

Nuclei were prepared from 1×10^8^ Ltk(−) cells stably co-transfected with TK plasmid and different PD1 constructs according to the protocol published by O'Neill and Turner [65]. DNA was purified using phenol-chloroform extraction followed by ethanol precipitation and subjected qPCR; amplification across the gene for all cell lines was corrected for copy number and normalized to the amplification from PD1cDNAint1-2/Sac clone using the -100 primer set.

### Chromatin Immunoprecipitation

Native chromatin immunoprecipitation (N-ChIP) was performed to analyze histones modifications according to the protocol published by O'Neill and Turner [65] with the addition of preclearing step prior to immunoprecipitation with 75 µl of salmon sperm DNA/protein A agarose beads (Upstate, NY) for one hour at 4°C. Antibodies that were used for detecting histones modifications are: anti dimethyl Histone H3 Lys4 (Upstate, 07-030), anti acetyl Histone H3 Lys 9/14 (Upstate, 06-599), anti dimethyl Histone H3 Lys9 (upstate, 07-441), anti Histone H2A.Z (Abcam, ab-4174). A no antibody control was included as well.

Cross-linked ChIP (X-ChIP) was used to test for binding factor according to protocol published by Upstate and using Upstate ChIP assay kit (Upstate,NY) with a few modifications. Briefly, 1×10^6^ Ltk(−) cells stably transfected with PD1cDNAint1-2/5-1 construct were crosslinked as detailed in the published protocol, followed by addition of 0.125 M glycine for five minutes at room temperature to stop the crosslinking reaction. Cells were washed twice with ice-cold PBS in the presence of protease inhibitors and then resuspended in SDS lysis buffer. The cells lysates were then sonicated in the presence of glass beads to give fragments size ranging from 1–0.3 Kb. ChIP proceeded according to the published protocol, using the following antibodies: anti CTCF (Santa cruz, sc15914x), anti USF1 (santa cruz, sc-229x), anti USF2 (Santa cruz, sc-861x), and anti CBP (santa cruz, sc-369), followed by reversal of crosslinking by over night incubation with NaCl at 65°C. A no antibody control was included as well.

### Quantitation of ChIP results

DNA immunoprecipitated by N-ChIP or X-ChIP reactions was analyzed by real time PCR using the following primers:

Promoter primers:

(-1000): sense: 5′ TACATATGAAACACTCCTGCTACCTTCC; antisense: 5′ CCAGTAAAGGTTGTATTCCATGA

(-600): sense: 5′ TGTGCGGGGCT TTTACATTTC; antisense: 5′ CACTGGAGGTTTATGTCTGCTTCTG

(-100): sense: 5′ CGCAACCTGTGTGGGAC; antisense: 5′ GGGTGGGTGGAGAGTTT

Coding region:

intron 1: sense: 5′ GAACAAGGCCGCTGCG; antisense: 5′ CAGCAGA GTCGCACCTTC

Exon 5; sense: 5′ CAGACCCTGCTCAGCCC 3′; antisense: 5′ G GGTGGGTGGAGAGTTT

3′ end:

3′UTR(1): sense: 5′ TCTGTGTTCCTATGAGCATCC; antisense: 5′GAACAC AGGTCAGGGTGAG

3′UTR(2): sense: 5′CTGACATCTCCATCCTTACT; antisense: 5′ CCTCATGGCACCAATTAGAA

3′UTR(3): sense: 5′ TGCCATGAGGAGTTGAGG GGATAATAAA; antisense: 5′ GGATTCTGGAAGGTTCTCA

3′distal(1): sense: 5′ ATTCAGGAGCTTGGTTAGCAA; antisense: 5′ GCCAAATACA AACACAGAATAAA

3′distal(2): sense: 5′ CCTCCTTCCAGAGATGTTTA; antisense: 5′ ACCTCCATGTGCCGTGAGT

3′distal(3): sense: 5′ GGGTCTAATCAGAGCTACAG; antisense: 5′ AGCATTGCTGTGTGTTGTGG

Quantitative real-time PCR was performed using ABI7900 with SYBR green real time PCR kit (Applied Biosystems). Results were calculated as percentage bound/input DNA as compared to no antibody control.

### CpG methylation

Genomic DNA was prepared from Ltk(−) clones stably transfected with PD1cDNAint1-2/Bam, PD1/Sac and PD1/polyA. The DNA was digested with HpaII (CpG methylation sensitive) or Msp I (CpG methylation insensitive). After digestion, the DNA was precipitated and a promoter region spanning two HpaII/Msp I restriction sites was amplified in a real time qPCR reaction using (−100) primers (see above). Results were calculated as percentage PCR product of non-digested DNA amplified with the same primers.

## Supporting Information

Figure S1 Tissue expression pattern of PD1cDNAint1-2/Bam transgenic mice parallels that of PD1/Bam transgenic mice. RNA was isolated from the indicated tissues and assessed by qPCR for the levels of PD1 specific RNA using a primer that spans exons 2–3. RNA levels were quantitated relative to an 18S rRNA internal control.
(0.01 MB PDF)

Figure S2 Loss of PD1 expression in the absence of 3′ intergenic sequences is not associated with promoter CpG methylation. DNA was isolated from L cell clones stably transfected with PD1cDNAint1-2/Bam, PD1cDNAint1-2/Sac and PD1cDNAint1-2/polyA and maintained in the absence of HAT medium. Although all three clones retain the respective transgenes, only the PD1cDNAint1-2/Bam line expresses PD1. CpG methylation at the promoter in each of the clones was determined as described in Supplementary Experimental Procedures. Results show real time PCR amplification of PD1 promoter region after digestion with Hpa II or Msp I, relative to non-digested DNA. Results are representative of three independent clones from each construct.
(0.06 MB TIF)

Figure S3 3′ intergenic sequences do not affect nucleosomal organization of expressed PD1 genes in the presence of selective pressure. Nucleosomal occupancy was determined across the PD1 gene in L cell clones stably transfected with PD1cDNAint1-2/Bam, PD1cDNAint1-2/Sac and PD1cDNAint1-2/polyA and maintained in the presence of HAT medium. All three clones expressed the PD1 gene to comparable levels.
(1.27 MB TIF)

Table S1 (0.03 MB DOC)

Table S2 (0.03 MB DOC)

Table S3 (0.03 MB DOC)
