# Supplementary figures and images for: In Vivo Expression of MHC Class I Genes Depends on the Presence of a Downstream Barrier Element
Source: PLoS One. 2009 Aug 26;4(8):e6748. doi: 10.1371/journal.pone.0006748 (PMC2727697; doi:10.1371/journal.pone.0006748)

Figure S1

qRT-PCR Analysis of PD1cDNAint1-2/Bam

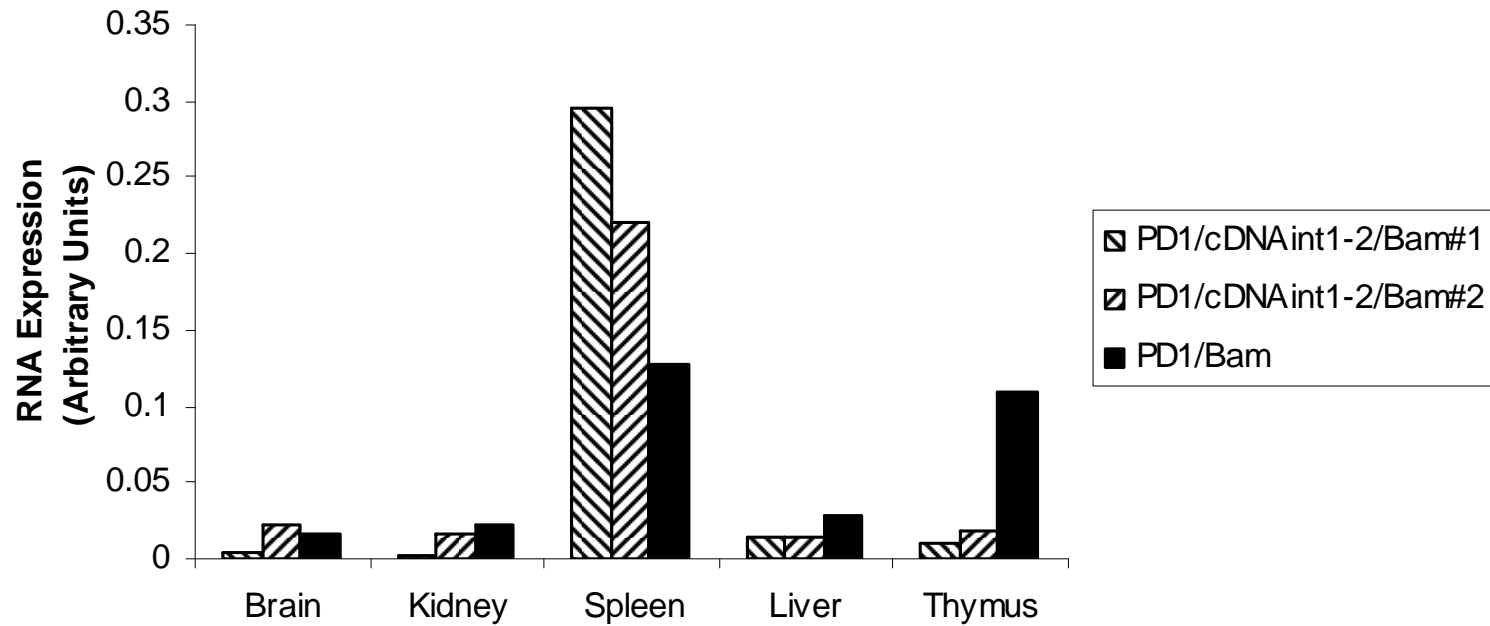

Supplement: Figure S1 — Tissue expression pattern of PD1cDNAint1-2/Bam transgenic mice parallels that of PD1/Bam transgenic mice. RNA was isolated from the indicated tissues and assessed by qPCR for the levels of PD1 specific RNA using a primer that spans exons 2–3. RNA levels were quantitated relative to an 18S rRNA internal control. (0.01 MB PDF) [file pone.0006748.s001.pdf]

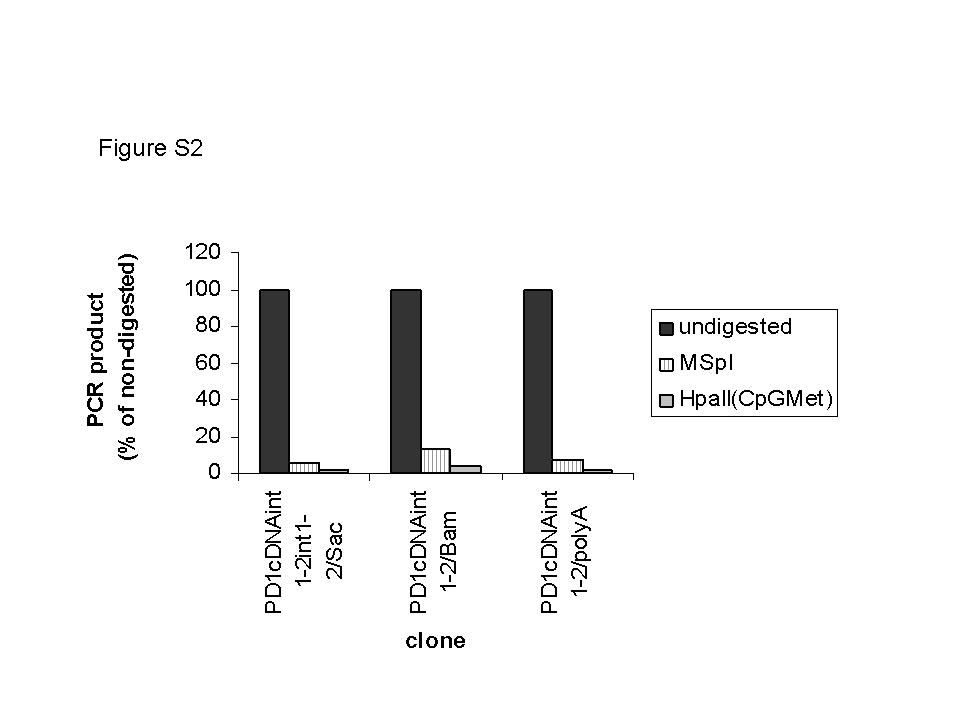

Supplement: Figure S2 — Loss of PD1 expression in the absence of 3′ intergenic sequences is not associated with promoter CpG methylation. DNA was isolated from L cell clones stably transfected with PD1cDNAint1-2/Bam, PD1cDNAint1-2/Sac and PD1cDNAint1-2/polyA and maintained in the absence of HAT medium. Although all three clones retain the respective transgenes, only the PD1cDNAint1-2/Bam line expresses PD1. CpG methylation at the promoter in each of the clones was determined as described in Supplementary Experimental Procedures. Results show real time PCR amplification of PD1 promoter region after digestion with Hpa II or Msp I, relative to non-digested DNA. Results are representative of three independent clones from each construct. (0.06 MB TIF) [file pone.0006748.s002.tif]

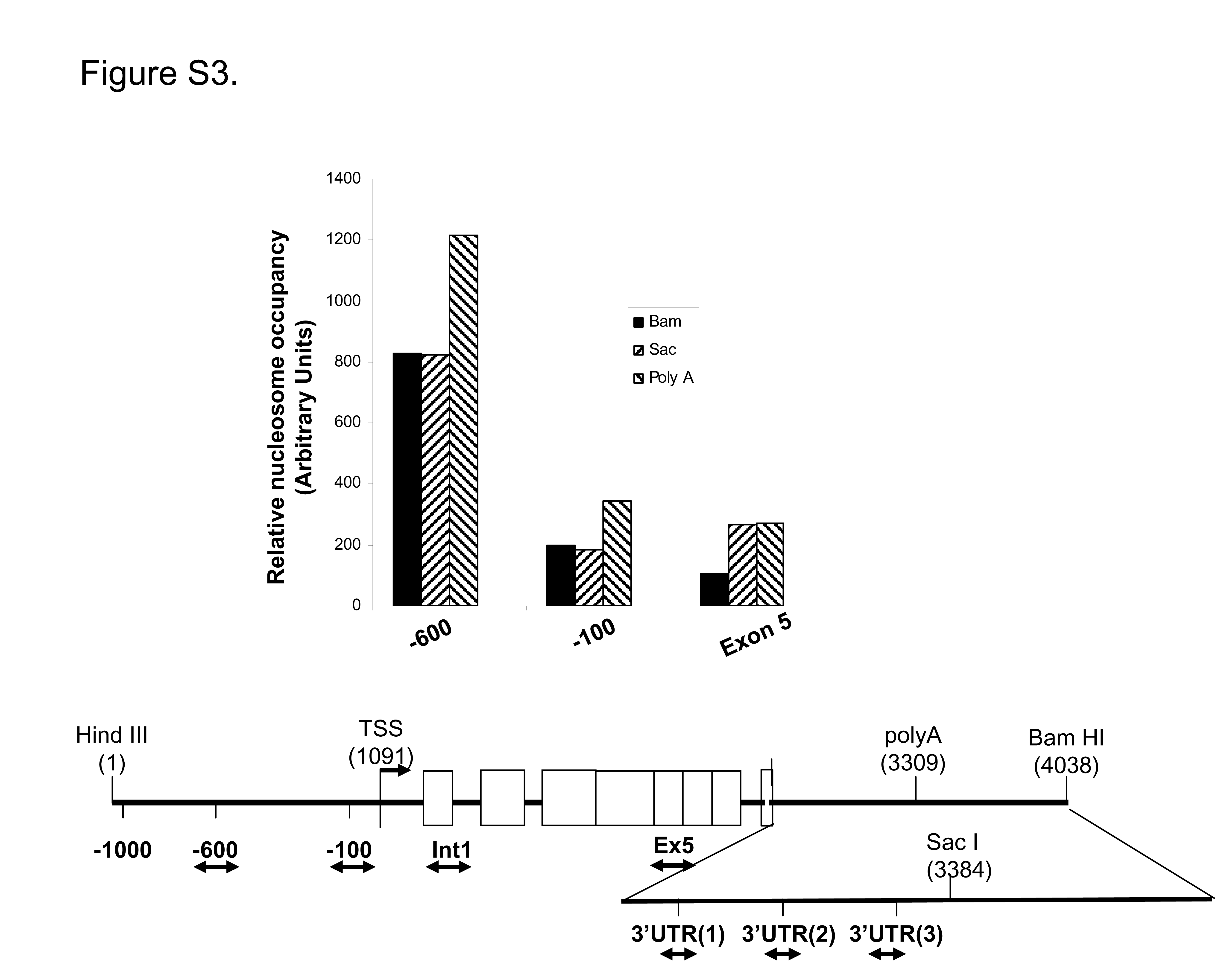

Supplement: Figure S3 — 3′ intergenic sequences do not affect nucleosomal organization of expressed PD1 genes in the presence of selective pressure. Nucleosomal occupancy was determined across the PD1 gene in L cell clones stably transfected with PD1cDNAint1-2/Bam, PD1cDNAint1-2/Sac and PD1cDNAint1-2/polyA and maintained in the presence of HAT medium. All three clones expressed the PD1 gene to comparable levels. (1.27 MB TIF) [file pone.0006748.s003.tif]
