# Supplementary material for: In Vivo Expression of MHC Class I Genes Depends on the Presence of a Downstream Barrier Element
Source: PLoS One. 2009 Aug 26;4(8):e6748. doi: 10.1371/journal.pone.0006748 (PMC2727697; doi:10.1371/journal.pone.0006748)
Supplement: Table S1 — (0.03 MB DOC) [file pone.0006748.s004.doc]

BamHI/Exon 7 Exon 8 3’ UTR

***GGATCCT****AGAG*GTGAGACCCTGGAGGGCC***TAG***ATGGGAGGGGGGTTGGGGCAGAGGGGGTGCCCTGGTGACGGGGATCTTTGAGGGGGTGGTTTGGAGCATGTGGGGCTGTTGAGCATGTCAGCCCTTCCTTGACTGACCTGTCCTGTTTCCTGATGATTTTCATTCCcagtgtGAGAcagctgCCTTGTGGGAACTGAGGGGACACAAGATTTGTTCACGTCCCACTTTGTGAATCCAGATCCCCTGACTCCTGTTTCTGCAGCTGCCCTCTGAAAGGGTCTGTGTTCCTATGAGCATTCCTGAGAGGAGGTTGGGACCCTGGCCCCCACGTTCCCCTCCTCACCCTGACCTGTGTTCTCTTCCATGATCCTCTTTCAGTTCTTGCAGTGGGAGCCGGCGGTGGGAGGGGGCACTGACATCTCCATCCTTACTTAACTTGAACTGCCCTGAGTAATGACTTCCTGTTGAAATTTGCTTTTTCTAATTGGTGCCATGAGGAGTTGAGGGG***ATAATAAAT***GAGAGATTTCCTATGTTTGAAAGAG*AAATAAA*TGGAAGGATTGAGAACCTTCCAGAATCcacatgTGTGCTGTGCTGTGTCAGTTCCGGATGGGATGTGAGGAGAGAAATCATGGACGGGCCTGTGCCCAGTGA***GAGCTC***AGGGCATCATGGGCTCGGTGTGGACACTCCCGAGGCTGGGTCACCTTTTCCCCTGTCCCTTTGTCCTTGTCCCTTCACGGAGAACTTTCTTCCACCAGGCCCTGTGATCTCAGGGACTGAGAAGTCCCTGGGCCTTGTCCTGTATTCAGGAGCTTGGTTAGCAAGGCCTCCTGTCACCAGGTAGCCTAGTCAGCCTTGGTCCCATCAGTAGTCCCTTTATTCTGTGTTTGTATTTGGCCTATTTAGATTTTTTTTTAAAATACGGACTTTTCATTTAcatgtgTGAAATTACAGTCTCATTTTTCTCTGGGTGTCCCTCGTCTGCAACAGCCACAACAGTCATTTGCCTGTCATTGTTCCcacatgTCCAGGGGGTCCCTGCTCAAAGATTCTCAGTGGTATCAAGAGATCCATTTTCAGCCTCATCCAGCTCTTGCCCTCCTTCCAGAGATGTTTACTGGATGCTTCTTTCTTTCTTTCTTTTTTTTTTTTTTTTGACTTTTCAGGGCCACACTCA

**Table S1. DNA sequence of 1.3 Kb 3’ BamHI Fragment Containing Exon 7, Intron 7, Exon 8, 3’ UTR and 3’ Intergenic Region of PD1 Gene**

BamHI, SacI and PolyA addition sites are italicized and in bold. Sequences homologous to E boxes are indicated in small letters.
