# Supplementary material for: In Vivo Expression of MHC Class I Genes Depends on the Presence of a Downstream Barrier Element
Source: PLoS One. 2009 Aug 26;4(8):e6748. doi: 10.1371/journal.pone.0006748 (PMC2727697; doi:10.1371/journal.pone.0006748)
Supplement: Table S2 — (0.03 MB DOC) [file pone.0006748.s005.doc]

| **Clone** | **Reduction of MHC class I surface expression**  **(%)** |
| --- | --- |
| 1  (2 copies) | 52% |
| 2  (2 copies) | 50% |
| 3 | 22% |
| 4 | 5% |
| 5  (4 copies) | 76% |
| 6 | 24% |
| 7 | 48% |
| 8 | 0% |

**Table S2. PD1cDNAint1-2/PolyA Clones without Barrier Element Lose Class I Expression in the Absence of Selective Pressure**

Levels of cell surface PD1 expression were assessed by FACS 140 days after removal from HAT medium. The results are expressed relative to the control cell population maintained in the presence of the selective medium.
