# Supplementary material for: In Vivo Expression of MHC Class I Genes Depends on the Presence of a Downstream Barrier Element
Source: PLoS One. 2009 Aug 26;4(8):e6748. doi: 10.1371/journal.pone.0006748 (PMC2727697; doi:10.1371/journal.pone.0006748)
Supplement: Table S3 — (0.03 MB DOC) [file pone.0006748.s006.doc]

|  | **Median FL** | |  |
| --- | --- | --- | --- |
| **Clone** | **DMEM** | **HAT** | **DMEM/HAT** |
| Ltk- | 64.4 | N/A | N/A |
| 1 | 30.4 | 60.4 | 0.503311 |
| 2 | 27.1 | 83.6 | 0.324163 |
| 3 | 57 | 82.2 | 0.693431 |
| 4 | 76.6 | 116 | 0.660345 |
| 5 | 28.3 | 126 | 0.224603 |
| 6 | 60.8 | 75.4 | 0.806366 |
| 7 | 51.4 | 82 | 0.626829 |
| 8 | 60.6 | 104 | 0.582692 |
| 9 | 33.7 | 39.5 | 0.853165 |

**Table S3. Deletion of 79 bp segment between polyA and SacI abrogates stable expression of the MHC class I gene in transfected L cell clones**.
